# Social dilemma in foraging behavior and evolution of cooperation by learning

**DOI:** 10.1038/s41598-023-49368-8

**Published:** 2023-12-11

**Authors:** Nahyeon Lee, Sunhee Chae, Seung Ki Baek, Hyeong-Chai Jeong

**Affiliations:** 1 Department of Physics and Astronomy, Sejong University, Seoul, 05006 Korea; 2 Department of Scientific Computing, Pukyong National University, Busan, 48513 Korea

**Keywords:** Evolution, Behavioural ecology, Evolutionary ecology, Population dynamics

## Abstract

We consider foraging behaviors in a two-dimensional continuum space and show that a cooperative chasing strategy can emerge in a social dilemma. Predators can use two different chasing strategies: A direct chasing strategy (DCS) and a group chasing strategy (GCS). The DCS is a selfish strategy with which a chaser moves straight toward the nearest prey, and the GCS is a cooperative strategy in the sense that the chaser chooses the chasing direction for the group at a cost of its own speed. A prey flees away from the nearest hazard, either a chaser or the boundary, within its recognition range. We check the capturing activities of each strategy and find a social dilemma between the two strategies because the GCS is more efficient for the group whereas the DCS is better individually. Using a series of numerical simulations, we further show that the cooperative strategy can proliferate when a learning process of nearby successful strategies is introduced.

## Introduction

Behavioral ecologists have investigated cooperative hunting in species like lions, dolphins, and wolves, as well as implemented robots^1–8^. Theoretically, extensive research has focused on learning and co-evolution of strategies of predators and prey^9–13^. Recently, statistical physicists are looking at the problem of group chase and escape with great interest so as to study systems of interacting active agents^14–25^. One of the findings in this direction is that the collective effect of group chase and escape is sometimes nontrivial, e.g., in that lazy chasers who more or less keep their own positions with performing random walks can improve the overall efficiency by helping other chasers to form the pincer movement spontaneously^22^.

How to catch a moving target efficiently in group is also an important topic in differential game theory^26–28^. Game theory has long considered a group hunt to be a prominent example of a social dilemma^29–31^. Real-world observations provide examples of these dilemmas, where individual interests during hunting often conflict with the collective aims of a group. For example, a single predator may prefer to chase an individual target separate from the group’s objective or might opt for a free-riding approach^32,33^.

Whereas group hunting is common in nature, agent-based simulations have shown that the transition from solitary to cooperative behavior requires a complex process and is often difficult to achieve^34,35^. Moreover, we currently lack empirical evidence of populations where both solitary and group hunting strategies coexist for the same target. Nonetheless, the absence of such evidence does not diminish the importance of studying models that encompass both solitary and group hunting behaviors. It is conceivable that in historical natural settings, populations practicing both strategies might have, due to evolutionary dynamics, converged or fixed onto one predominant approach-either solitary or group-based. Such evolutionary trajectories could elucidate the present-day rarity of populations exhibiting both strategies. Thus, understanding the emergence of group hunting strategies remains intriguing, especially when solitary hunting is individually favored, even though group hunting might be more effective collectively.

In the context of group hunting, a social dilemma arises when an individual’s cooperative behavior can benefit all the others in the group, whereas it may incur a cost to him or her. A similar dilemma is also found in soccer if players are rewarded only for scoring goals. This incentivizes players to prioritize individual success by shooting for the goal rather than passing to a better-positioned teammate, hindering the emergence of cooperative behaviors aimed at team success. Some might argue that a passing strategy could emerge through group selection^36^, whereby a team that uses a more cooperative and altruistic approach to scoring end up with scoring more goals on average and thus have a higher payoff. However, as long as group selection operates on a longer time scale than that of individual selection, it is hard to suppress the invasion of free-riders who receive passes but never make passes. Therefore, without a discriminator strategy such as passing only to those who have also passed to others, it will be challenging to maintain an altruistic passing strategy.

Note that the term ‘cooperative behavior’ in predator-prey models has been used with two related yet distinct meanings. In some literature, it refers to ‘coordinated behavior’ where predators or prey work together towards a common goal. However, this is not the exactly same as the concept of cooperation in game theory. In game theory, particularly in the context of social dilemmas, cooperation entails an agent paying a cost for the benefit of other agents. Although many papers have shown cooperative behaviors in the coordinated sense^37–40^, it has been difficult to demonstrate the game-theoretic cooperation in predator-prey models.

In this work, we consider a model in which the reward is exclusively given to chasers who successfully capture targets. We then propose and compare two chasing strategies for catching moving targets. One is a direct chasing strategy (DCS), moving straight to the nearest target. The other is a group chasing strategy (GCS), which takes into account other nearby chasers cooperatively at a cost of speed: GCS chasers are slower than DCS chasers because they have to recognize the position of their colleagues and calculate the direction for the group, leading to a cognitive load. Therefore, the hunting ability of GCS chasers decreases resulting in a cost to GCS chasers. By means of numerical simulation, we first find that the DCS is individually better, whereas the GCS shows better performance collectively, hence a social dilemma. Nonetheless, we will demonstrate that GCS can be adopted by the entire population of chasers, if each chaser learns the strategy of a nearby chaser that just caught a target. In other words, cooperation can emerge even without considering explicit mechanisms^41–46^ such as direct and indirect reciprocity or the long-term advantages of being part of an altruistic group.

The organization of this paper is as follows: The next section explains our model and numerical procedure. We present numerical results in “Results” Section: First, we compare the performance of the DCS with that of the GCS by simulating them one by one. Second, we check what happens if these two strategies coexist. Third, we include the learning process to see which strategy succeeds in fixation. Then, we discuss its implication in terms of evolution of cooperation. Finally, this work is summarized in “Summary and Discussion” Section.

## Model

We investigate an agent-based foraging behaviors in a two-dimensional disk of radius *R*. The population is composed of $N_{\text {T}}$ targets and $N_{\text {C}}$ chasers, where chasers can adopt either a DCS or GCS strategy.

We consider three different scenarios: Model A, Model B, and Model C, to evaluate and compare the effectiveness of both DCS and GCS chasing strategies, and to study their evolution.

In Model A, we assume that all chasers employ a homogeneous strategy, resulting in two distinct populations: one consisting of targets and DCS chasers and the other consisting of targets and GCS chasers. By contrast, in Model B, we assume the coexistence of both DCS and GCS chasers in a population, with half of the chasers using each strategy, but without any learning process. Last, Model C is similar to Model B but includes a learning process, where each chaser can adopt the strategy of a nearby chaser that successfully caught a target.

### Targets

Let us begin by describing the movement of a target. Each specific target is indexed by $T_i$ with $i\in [1,N_{\text {T}}]$, and similarly, each specific chaser is indexed by $C_i$ with $i\in [1,N_{\text {C}}]$. Every target moves at a speed of $v_{_\text {T}}$, and it regards chasers and the boundary of the disk as hazardous when they are within a certain distance of $r_{_{\!\text {haz}}}$. If a target perceives no hazard nearby, it moves in random directions. However, if it finds at least one chaser or the boundary within $r_{_{\!\text {haz}}}$, it moves right away from it by taking the following direction:

1 $$\begin{aligned} \hat{v}_{_{T_i}}= \frac{\textbf{r}_{_{T_i}}-\textbf{r}_{_{H_i}}}{\left| \textbf{r}_{_{T_i}}-\textbf{r}_{_{H_i}}\right| }, \end{aligned}$$

where $\textbf{r}_{_{H_i}}$ is the position of the nearest hazard of $T_i$. Targets move straight in the specified direction for a duration of $\delta t$ representing inertial motion.

### Chasers

**Figure 1:**
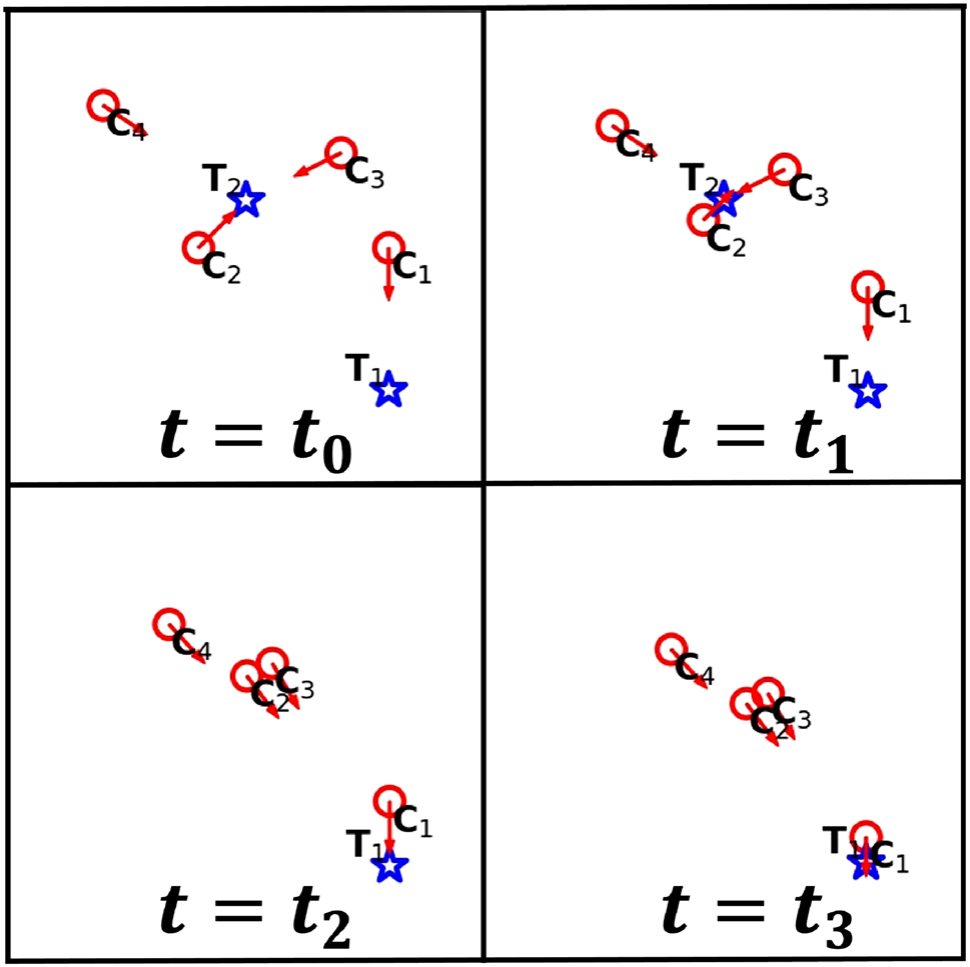
Two stationary targets (blue stars) and four DCS chasers (red circles). At time $t_0$, chaser $C_1$ moves towards target $T_1$, whereas the other chasers move towards target $T_2$. Once target $T_2$ is caught (at time $t_1$), all the chasers then pursue target $T_1$ (at times $t_2$ and $t_3$). Here, we use stationary targets to explain the motion of chasers clearly but in simulations, targets move away and are replaced at a random position after capture.

**Figure 2:**
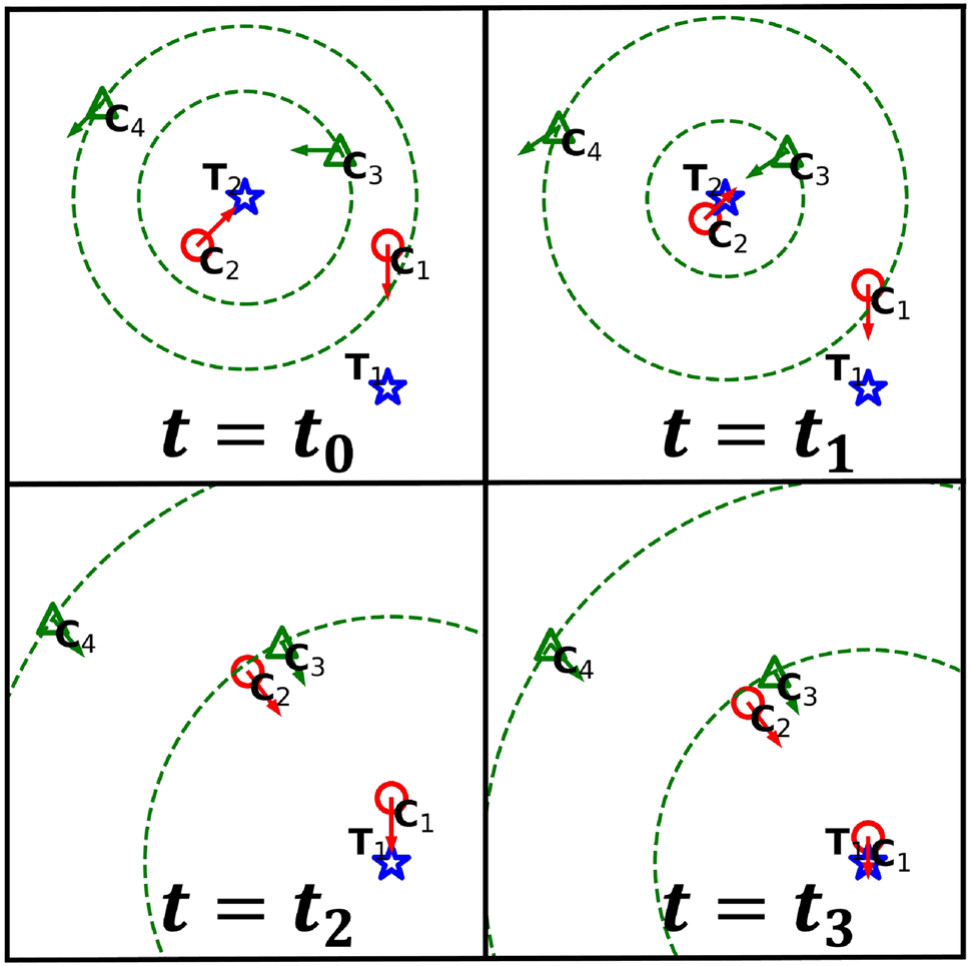
Two stationary targets (blue stars), two DCS chasers (red circles), and two GCS chasers (green triangles). Each GCS chaser chooses the nearest target and takes into account other chasers that are closer to the target (in the green dotted circles). At time $t_0$, $C_3$ chooses its movement direction so that the center of $C_2$ and $C_3$ moves toward $T_2$. Likewise, $C_4$ takes into account the centers of all four chasers in determining its movement direction, resulting in it moving away from $T_2$. Once target $T_2$ is caught (at time $t_1$), $T_1$ becomes the new target of GCS chasers $C_3$ and $C_4$. At times $t_2$ and $t_3$, GCS chasers consider the positions of other chasers within the green dotted circles around $T_1$ in order to determine their directions of movement.

Now we consider a chaser’s movement, specifically in hunting mode (for an explanation of hunting mode, see below). If a chaser uses the DCS, it moves straight to the nearest target. More precisely, if $T_j$ is the nearest target from a DCS chaser $C_i$, the chaser moves in the direction of

2 $$\begin{aligned} \hat{v}_{_{C_i}}= \frac{\textbf{r}_{_{T_j}}-\textbf{r}_{_{C_i}}}{\left| \textbf{r}_{_{T_j}}-\textbf{r}_{_{C_i}}\right| } \end{aligned}$$

at a speed of $v_{_\text {D}}$. Fig. 1 demonstrates the movement of DCS chasers when the targets are stationary. This illustration is provided to clarify their motion. In the actual simulations, the targets are moving away from the chasers as previously described. Additionally, after a target is captured, a new target is randomly added to the simulation.

The GCS is a more sophisticated rule, taking into account other nearby chasers as well as the position of the nearest target as illustrated in Fig. 2. A GCS chaser aims to place the nearest target at the center of all chasers (including itself) that are not farther than itself to the target. It may move *away* from the target if necessary to achieve this goal.

Let $T_k$ be the nearest target from a GCS chaser $C_i$. Their distance is denoted as $d_{C_i} \equiv \left| \textbf{r}_{_{T_k}}- \textbf{r}_{_{C_i}}\right|$. We also define $\Gamma _i$ as the group of chasers, excluding $C_i$, that are within a distance of $d_{C_i}$ from $T_k$. The GCS chaser $C_i$ moves in the following direction:

3 $$\begin{aligned} \hat{v}_{_{C_i}}= \frac{\textbf{X}_{_{C_i}}- \textbf{r}_{_{C_i}}}{\left| \textbf{X}_{_{C_i}}- \textbf{r}_{_{C_i}}\right| }, \end{aligned}$$

where $\textbf{X}_{_{C_i}}$ is defined as

4 $$\begin{aligned} \textbf{X}_{_{C_i}}\equiv \textbf{r}_{_{T_k}}+ \sum _{j\in \Gamma _i} \left( \textbf{r}_{_{T_k}}- \textbf{r}_{_{C_j}}\right) = (n_i+1)\, \textbf{r}_{_{T_k}}- \sum _{j\in \Gamma _i} \textbf{r}_{_{C_j}}, \end{aligned}$$

where $n_i \equiv \left| \Gamma _i \right|$. If $C_i$ is the nearest chaser from $T_k$, Eq. (3) reduces to Eq. (2) because $\Gamma _i = \emptyset$ and $n_i=0$. A GCS chaser moves at a speed of $v_{_\text {G}}$, and we assume that $v_{_\text {G}}< v_{_\text {D}}$ to penalize chasers for the cognitive load required by the GCS. We also assume that a target moves faster than a chaser, i.e., $v_{_\text {C}}<v_{_\text {T}}$ where $v_{_\text {C}}$ is $v_{_\text {D}}$ or $v_{_\text {G}}$ depending on the chaser’s strategy. This is because a slower prey would be easily captured without cooperative hunting in the first place^23^. The inequalities in the speeds are summarized as follows:

5 $$\begin{aligned} v_{_\text {G}}< v_{_\text {D}}< v_{_\text {T}}. \end{aligned}$$

Moreover, a chaser can change its strategy between the DCS and GCS in the following way: When a chaser succeeds in catching a target, other chasers within the distance of $r_{_{\!\text {learn}}}$ adopt the focal chaser’s strategy.

### Hunting and rest

A chaser has two modes: One is the hunting mode and the other is the rest mode. In the hunting mode, the chaser’s moving direction is determined by its strategy as defined above. In the rest mode, on the other hand, the chaser moves in random directions. The maximum amount of time in the hunting mode is denoted as $T_\text {hunt}$: If a chaser has been hunting continually for a period of $t_{\text {hunt}}$, the probability to switching to the rest mode is set as follows:

6 $$\begin{aligned} p_{_\text {rest}}= \min \left( \frac{t_{\text {hunt}}}{T_\text {hunt}}, 1\right) . \end{aligned}$$

Likewise, if a chaser has rested for $t_{\text {rest}}$, it starts the hunting mode again with probability

7 $$\begin{aligned} p_{_\text {hunt}}= \min \left( \frac{t_{\text {rest}}}{T_\text {rest}}, 1\right) , \end{aligned}$$

where $T_\text {rest}$ is the maximum amount of time in the rest mode.

### Simulation procedure^47^

**Table 1:** Parameters employed in the simulations.

Symbol	Definition	Value
*R*	Disk radius	500
$N_{\text {C}}$	Number of chasers	100
$N_{\text {T}}$	Number of targets	50
$v_{_\text {G}}$	A GCS chaser’s speed	0.07
$v_{_\text {T}}$	A target’s speed	0.1
$v_{_\text {D}}$	A DCS chaser’s speed	[0.072, 0.098]
$\delta {t}$	Time interval	1
$r_{_{\!\text {min}}}$	Minimal distance between chasers or targets	0.15
$r_{_{\!\text {haz}}}$	Maximal distance for recognizing hazard	50
$r_{_{\!\text {learn}}}$	Maximal distance for learning	200
$T_\text {hunt}$	Maximum amount of time for the hunting mode	$10^3$
$T_\text {rest}$	Maximum amount of time for the rest mode	$10^3$

Our numerical simulation goes as follows:

1. Randomly distribute $N_{\text {C}}$ chasers and $N_{\text {T}}$ targets in a two-dimensional disk of radius *R*.
2. Calculate the new attempted positions for all chasers.
  Determine the chaser’s mode using Eqs. (6) and (7).
  In the rest mode, the attempted position is calculated in a random direction.
  In the hunting mode, the attempted position depends on the nearest target’s position. If this target is within a distance of $v_{_\text {C}}\delta {t}$ from the chaser, the attempted position is given by the target’s position. Otherwise, the chaser’s attempted position is determined by moving $v_{_\text {C}}\delta {t}$ in the direction specified by either Eqs. (2) or (3).
3. Calculate the new attempted position for all targets.
  When the nearest chaser or boundary is within distance of $r_{_{\!\text {haz}}}$, the target’s attempted position is calculated by moving $v_{_\text {T}}\delta {t}$ in the direction specified by Eq. (1). Otherwise, the attempted position is calculated in a random direction.
4. Update the chasers’ position.
  Choose a chaser in random order, and update its position to the attempted position unless another chaser exists within distance $r_{_{\!\text {min}}}$ from it.
5. Update the targets’ position.
  Randomly select a target.
  If the target is located at the updated position of the chasers, it is considered captured and removed. A new target is then added at a random position separated by at least $r_{_{\!\text {min}}}$ from any other agent in the habitat.
  Otherwise, update its position to the attempted position unless another target exists within distance $r_{_{\!\text {min}}}$ from it.
6. (Model C only) Update the chasers’ strategies.
  Update the strategies of chasers within distance $r_{_{\!\text {learn}}}$ from the captured target to the strategy of the chaser who caught the target.
7. Increase time *t* by $\delta {t}$.
8. Repeat the above steps from 2 to 7, until *t* exceeds $T_\text {max}=10^6$.

The parameters in our simulation are listed in Table 1. The reasoning behind our procedures and parameters is as follows. For process 1, we select a disk-shaped habitat instead of a square habitat because a high proportion of targets are observed to become trapped in corners in the latter. We believe that the shape of the habitat does not have a significant impact on the results as long as it does not contain singular points such as corners. In process 2, the inclusion of a rest mode is implemented to reflect the reality that a chaser cannot hunt the target indefinitely. Furthermore, the absence of a rest mode resulted in unnatural movement patterns for chasers, such as constantly circling the edge of the habitat. Here, the distance $v\delta {t}$ is the basic step distance an agent travels in the simulation. It is given by $v_{_\text {D}}\delta {t}$ for the DCS and $v_{_\text {G}}\delta {t}$ for the GCS. The time interval $\delta {t}$ should be interpreted as the minimum time of linear motion due to inertia, rather than the finite time required for numerical integration. In processes 5, we introduce $r_{_{\!\text {min}}}$ to prevent collisions between members of the same species. The minimum separation distance, $r_\text {min} = 0.15$ exceeds the basic step distance, $v\,\delta t$, which is less than or equal to 0.1.

## Results

In this section, we first present numerical evidence that the GCS is better for the group, but the DCS is better for individuals using Models A and B, indicating a social dilemma between the two strategies. In Model C, we demonstrate that the whole chaser population can evolve towards the GCS despite this dilemma through a simple dynamics of learning from successful chasers in the neighborhood. The figures in this section are based on average results over 1000 independent simulations with different random initial distributions of targets and chasers. The video of a single simulation for each model can be found on the platform of YouTube^48^.

### Model A: DCS and GCS population comparison

**Figure 3:**
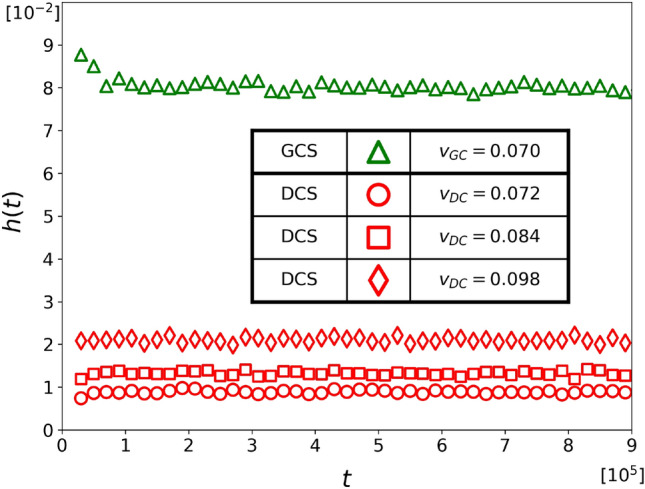
The hunting performance comparison of two chasing strategies, represented by $h(t)$, during a time interval of $\left( {t-\Delta {t}/2,\ t+\Delta {t}/2} \right)$, where $\Delta {t}=2\times 10^4$. The green triangles represent Population G, which consists of $N_{\text {C}}=100$ GCS chasers with a speed of $v_{_\text {G}}=0.07$, while the red circles, squares, and diamonds represent Population D, which consists of $N_{\text {C}}=100$ DCS chasers with speeds of $v_{_\text {D}}=0.072$, $v_{_\text {D}}=0.084$, and 0.098 respectively. Despite the fact that chasers in Population G are slower, they capture more targets. In all cases, the target speed is set at $v_{_\text {T}}=0.1$.

Model A assumes that all chasers in a population employ the same strategy, leading to the formation of two distinct populations labeled as G and D. Population G has $N_{\text {C}}=100$ GCS chasers, while population D has $N_{\text {C}}=100$ DCS chasers. Initially, both populations have $N_{\text {T}}=50$ targets, and this number remains constant because a new target is introduced to the population whenever a chaser captures a target. For population D, we consider three different speeds of chasers: $v_{_\text {D}}=0.072$, $v_{_\text {D}}=0.084$, and 0.098. The speed of targets is set to $v_{_\text {T}}= 0.1$. For population G, we study only one case with a speed of $v_{_\text {G}}= 0.07$. This means that the GCS chasers in population G are always slower than the DCS chasers in any population D. Figure 3 shows $h(t)$, hunting performance at time *t* for both population G and population D which is defined by the number of targets captured during $\left( {t-\Delta {t}/2,\ t+\Delta {t}/2} \right)$ per chaser with $\Delta {t}=2\times 10^4$. The data is obtained by averaging 5000 independent Monte Carlo simulations with different initial configurations. This result demonstrates that the GCS performs better than the DCS when applied globally, as mutual cooperation leads to better payoffs than mutual defection.

### Model B: coexistence of DCS and GCS without learning

We now study the relative performance of the DCS and GCS chasers when both strategies coexist in a population. Initially, we randomly distribute $N_{\text {C}}/2=50$ GCS chasers, 50 DCS chasers, and $N_{\text {T}}=50$ targets in the habitat. Although the total number of chasers in the population using each strategy remains constant, the number of chasers pursuing a specific target may change over time. Therefore, the relative performance of the two strategies depends on the specific composition of chasers around the target at any given time. To analyze this dependency, we introduce the concept of ‘set’, which means a group of chasers that are pursuing the same target. Sets are divided into two categories, i.e., homogeneous ones, which consist chasers of only one strategy, and heterogeneous ones, which contain chasers of both strategies.

**Figure 4:**
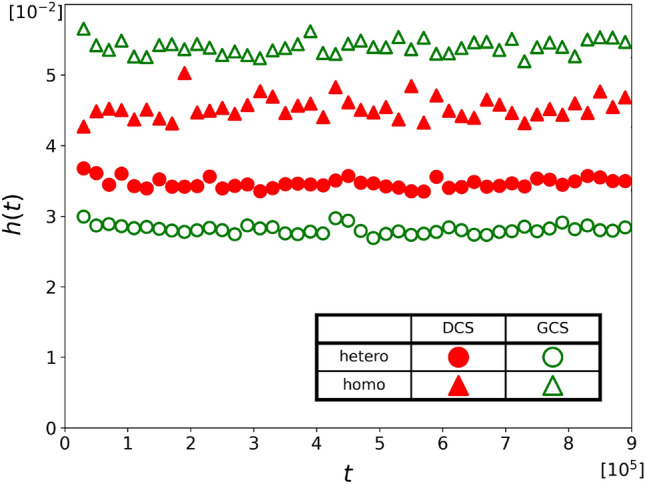
Hunting performance $h(t)$ of the GCS (green symbols) and that of the DCS (red symbols) in the heterogeneous sets (circles) and the homogeneous sets (triangles) for $v_{_\text {D}}=0.082$ with $v_{_\text {G}}= 0.070$ and $v_{_\text {T}}= 0.1$.

We compare the hunting performance of chasers in both homogeneous and heterogeneous sets separately. As before, we use the hunting performance $h(t)$, which represents the average number of captured targets per chaser within a time interval of $\left( {t-\Delta {t}/2,\ t+\Delta {t}/2} \right)$. However, differently from Model A, we must now track the numbers of GCS and DCS chasers in each set, in addition to the number of captured targets because the numbers of GCS and DCS chasers are also changing over time. Figure 4 displays the hunting performance of GCS and DCS chasers. As expected from the results of Model A, in homogeneous sets, the GCS chasers (green triangles) demonstrate superior hunting performance compared to DCS chasers (red triangles). However, in heterogeneous sets, the performance of DCS chasers (red circles) surpasses that of GCS chasers (green circles). If we use $h(t)$ as a metric of the payoff, it can be concluded that GCS chasers are better off than DCS chasers in homogeneous sets, but the DCS chasers have greater payoffs than the GCS in heterogeneous sets.

We observe that the hunting performance remains constant after an initial period of time $t<t_\text {ini}\approx 2\times 10^5$. We regard it as a steady state, characterized by the hunting performance remaining stable within statistical fluctuations. To ensure accuracy, we measure the average hunting performance between time intervals $5\times 10^5 \le t \le 9\times 10^5$ to determine the hunting performance in the steady state, $h^\text {ss}$.

**Figure 5:**
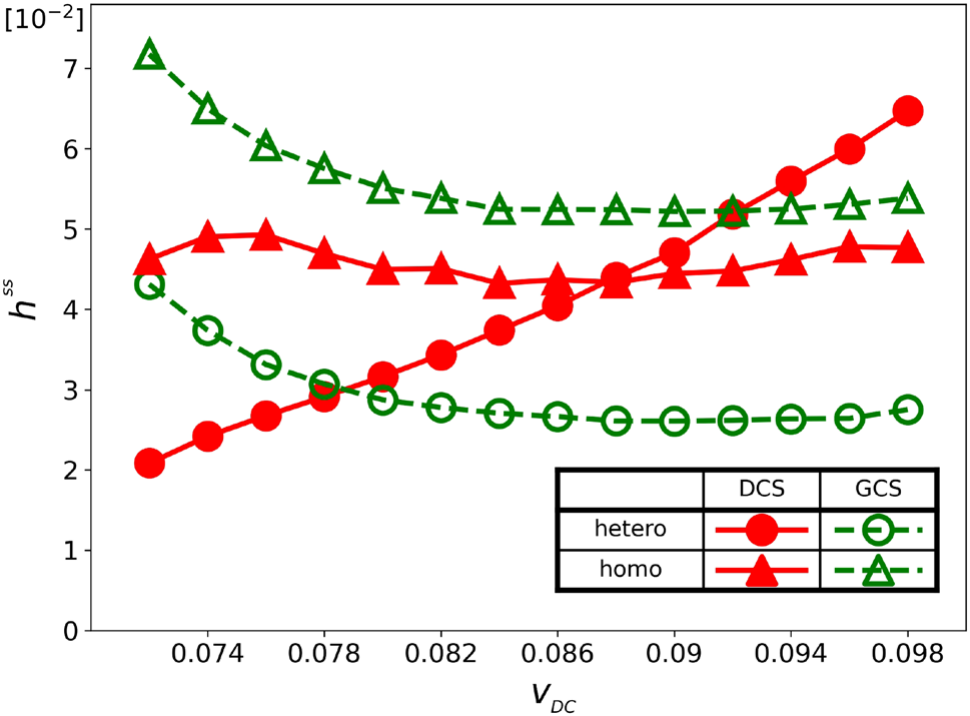
Hunting performance in steady state, $h^\text {ss}$ is plotted against the speed of DCS chasers, $v_{_\text {D}}$ for $v_{_\text {D}}=0.072, 0.074, \cdots , 0.098$. In heterogeneous sets (circles), DCS (red symbols) captures more targets for $v_{_\text {D}}\gtrsim 0.08$ while the GCS (green symbols) outperforms the DCS in homogeneous sets (triangles) for all $v_{_\text {D}}$ considered in here. In all cases, $v_{_\text {G}}= 0.07$ and $v_{_\text {T}}= 0.1$.

In Fig. 5, we present $h^\text {\,ss}$, hunting performance in steady state as a function of $v_{_\text {D}}$. The figure shows that GCS chasers outperform DCS chasers in homogeneous sets, while DCS chasers are better in catching targets in heterogeneous sets when $v_{_\text {D}}\gtrsim 0.08$. Yet, this does not necessarily imply a social dilemma in the strict sense of game theory. If a chaser in a homogeneous set of GCS chasers changes its strategy to DCS, the set becomes heterogeneous. Therefore, to understand the strategy evolution, we need to compare the payoffs of the strategies in sets with different numbers of GCS and DCS chasers. For this reason, we classify sets based on the numbers of GCS and DCS chasers present in each set and denote the set as $(n_{_\text {G}},n_{_\text {D}})$-set when it has $n_{_\text {G}}$ GCS and $n_{_\text {D}}$ DCS chasers. We then count the number of $(n_{_\text {G}},n_{_\text {D}})$-sets in the steady state every $\delta t$, and sum up the total observed $(n_{_\text {G}},n_{_\text {D}})$-sets to obtain $N(n_{_G},n_{_D})$. We also count the total numbers of targets captured by GCS ($H_{\text {G}}(n_{_\text {G}},n_{_\text {D}})$) and DCS chasers ($H_{\text {D}}(n_{_\text {G}},n_{_\text {D}})$) in $(n_{_\text {G}},n_{_\text {D}})$-sets during this steady state. Then we define the fitness ${f_{_G}(n_{_G},n_{_D})}$ and ${f_{_D}(n_{_G},n_{_D})}$ of GCS and DCS chasers in $(n_{_\text {G}},n_{_\text {D}})$-sets as their relative hunting performances,

8 $$\begin{aligned} {f_{_G}(n_{_G},n_{_D})}= & {} \frac{H_{\text {G}}(n_{_\text {G}},n_{_\text {D}})/H^\text {total}}{N_{\text {G}}(n_{_G},n_{_D})/N_{\text {C}}^\text {total}}, \nonumber \\ {f_{_D}(n_{_G},n_{_D})}= & {} \frac{H_{\text {D}}(n_{_\text {G}},n_{_\text {D}})/H^\text {total}}{N_{\text {D}}(n_{_G},n_{_D})/N_{\text {C}}^\text {total}} \end{aligned}$$

where $N_{\text {G}}(n_{_G},n_{_D})= n_{_\text {G}}N(n_{_G},n_{_D})$ and $N_{\text {D}}(n_{_G},n_{_D})= n_{_\text {D}}N(n_{_G},n_{_D})$ are the total numbers of GCS and DCS chasers counted in $(n_{_\text {G}},n_{_\text {D}})$-sets, respectively, $H^\text {total}$ is the total number of targets caught, and $N_C^\text {total}$ is the total number of chasers measured in the steady state. Note that the values of ${f_{_G}(n_{_G},n_{_D})}$ and ${f_{_D}(n_{_G},n_{_D})}$ will be equal to one if all chasers have the same hunting ability regardless of their strategy and set, because $H^\text {total}=\sum _{n_{_\text {G}},n_{_\text {D}}} \left[ {H_{\text {G}}(n_{_\text {G}},n_{_\text {D}})+H_{\text {D}}(n_{_\text {G}},n_{_\text {D}})} \right]$ and $N_C^\text {total}=\sum _{n_{_\text {G}},n_{_\text {D}}} \left[ {N_{\text {G}}(n_{_G},n_{_D})+N_{\text {D}}(n_{_G},n_{_D})} \right]$.

**Figure 6:**
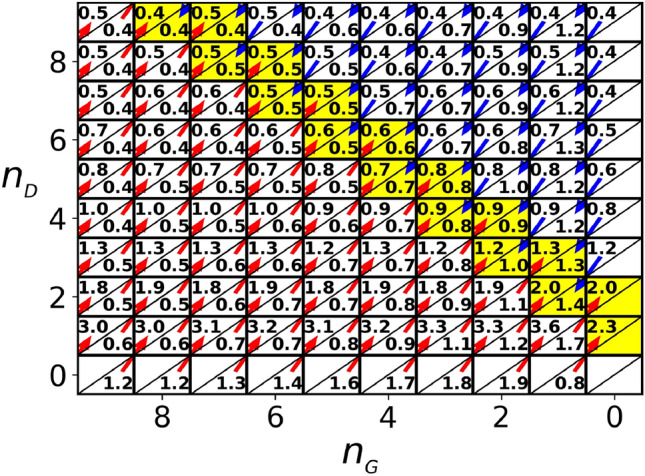
Average fitness of GCS, ${f_{_G}(n_{_G},n_{_D})}$ and DCS, ${f_{_D}(n_{_G},n_{_D})}$ for all combinations of $n_{_\text {G}}$ and $n_{_\text {D}}$ for $0\le n_{_\text {G}}\le 9$ and $0\le n_{_\text {D}}\le 9$. The *x*-axis displays $n_{_\text {G}}$ in descending order and the *y*-axis shows $n_{_\text {D}}$ in ascending order, with the diagonal axis indicating equal numbers of chasers ($n_{_\text {G}}+ n_{_\text {D}}$). ${f_{_G}(n_{_G},n_{_D})}$ and ${f_{_D}(n_{_G},n_{_D})}$ values are represented by numbers in the lower right and upper left halves of the $(n_{_\text {G}},n_{_\text {D}})$ cells respectively. Red (blue) arrows indicate a GCS (DCS) chaser can improve its fitness by switching strategies. The simulation uses $v_{_\text {D}}= 0.082$, $v_{_\text {G}}=0.07$ and $v_{_\text {T}}=0.1$.

Figure 6 displays the average fitness of GCS and DCS chasers for $v_{_\text {D}}= 0.082$ (with $v_{_\text {G}}=0.07$) in $(n_{_\text {G}},n_{_\text {D}})$-sets. In principle, the number of chasers in a set can be up to 50 for each type because all GCS or DCS chasers in the population can chase a single target. However, for clarity, the figure only shows the fitness for sets with small numbers of chasers ($0\le n_{_\text {G}}\le 9$ and $0\le n_{_\text {D}}\le 9$), because sets with large numbers of chasers are less common. In each cell, the average fitness of GCS and DCS chasers are represented by numbers at the lower right and the upper left halves, respectively. Note that the *x*-axis shows $n_{_\text {G}}$ in the descending order while the *y*-axis shows $n_{_\text {D}}$ in ascending order, so that the cells along the diagonal axis have equal numbers of chasers. If a GCS chaser in a $(n_{_\text {G}},n_{_\text {D}})$-set switches its strategy to DCS, the set becomes a $(n_{_\text {G}}-1,n_{_\text {D}}+1)$ set. Therefore, we need to compare ${f_{_G}(n_{_G},n_{_D})}$ and $f_{_\text {D}}(n_{_\text {G}}\!-\!1,n_{_\text {D}}\!+\!1)$ so as to study the strategy evolution. Red arrows are shown from the lower right halves of the $(n_{_\text {G}},n_{_\text {D}})$ cells to the upper left halves of the $(n_{_\text {G}}-1,n_{_\text {D}}+1)$ cells when ${f_{_G}(n_{_G},n_{_D})}< f_{_\text {D}}(n_{_\text {G}}\!-\!1,n_{_\text {D}}\!+\!1)$. These arrows indicate that a GCS chaser can improve its fitness by switching its strategy to DCS. Similarly, blue arrows indicate instances where a DCS chaser can improve its fitness by switching its strategy because ${f_{_D}(n_{_G},n_{_D})}< f_{_\text {G}}(n_{_\text {G}}\!+\!1,n_{_\text {D}}\!-\!1)$.

In Model B, chasers are not allowed to change their strategies. However, if they could, they would choose the direction indicated by the arrows to increase their payoffs when they stay in the same cell. The yellow shaded cells in Figure 6 correspond to these arrow directions and represent the Nash equilibrium in the sense that a chaser in a yellow cell cannot increase its payoff by switching its strategy when a fixed number of chasers, $n_c=n_{_\text {G}}+n_{_\text {D}}$, are chasing a target. For instance, consider chasers in a $(n_{_\text {G}},n_{_\text {D}})=(1,2)$-set. If the GCS chaser changes its strategy to DCS, the set becomes a (0,3)-set, and the fitness of the chaser reduces to 1.2 from 1.4. If a DCS chaser in the $(n_{_\text {G}},n_{_\text {D}})=(1,2)$-set switches to GCS, the set becomes a (2,1)-set, and its fitness reduces to 1.2 from 2.0. It is worth noting that the yellow cells have the property that their $n_{_\text {D}}$ value is slightly greater than their $n_{_\text {G}}$ value. This indicates that there would be more DCS chasers than GCS chasers in every set if chasers could change their own strategies to maximize the payoffs.

Up to now, our comparison of DCS and GCS strategies has been based on average target captures. However, if more individuals in one strategy consistently fail to catch any targets, its individual superiority, despite its higher average, becomes debatable. To investigate this, we analyzed the hunting variation between DCS and GCS. We find no significant disparities in the variation between the two strategies. Additionally, we observe a strong correlation between a decline in average performance and an increased fraction of unsuccessful chasers. This suggests that higher performance can also serve as a survival marker amid such variations.

### Model C: coexistence of DCS and GCS with learning

In this model with learning process, chasers update their own strategies based on successful captures. Whenever a target is captured by a chaser, the strategies of other chasers within a certain distance $r_{_{\!\text {learn}}}$ from the captured target are updated to the strategy of the successful chaser. As a result, the proportion of chasers using DCS and GCS strategies change over time and the population will eventually consist of only one type of chasers, which is referred to as “fixation”.

**Figure 7:**
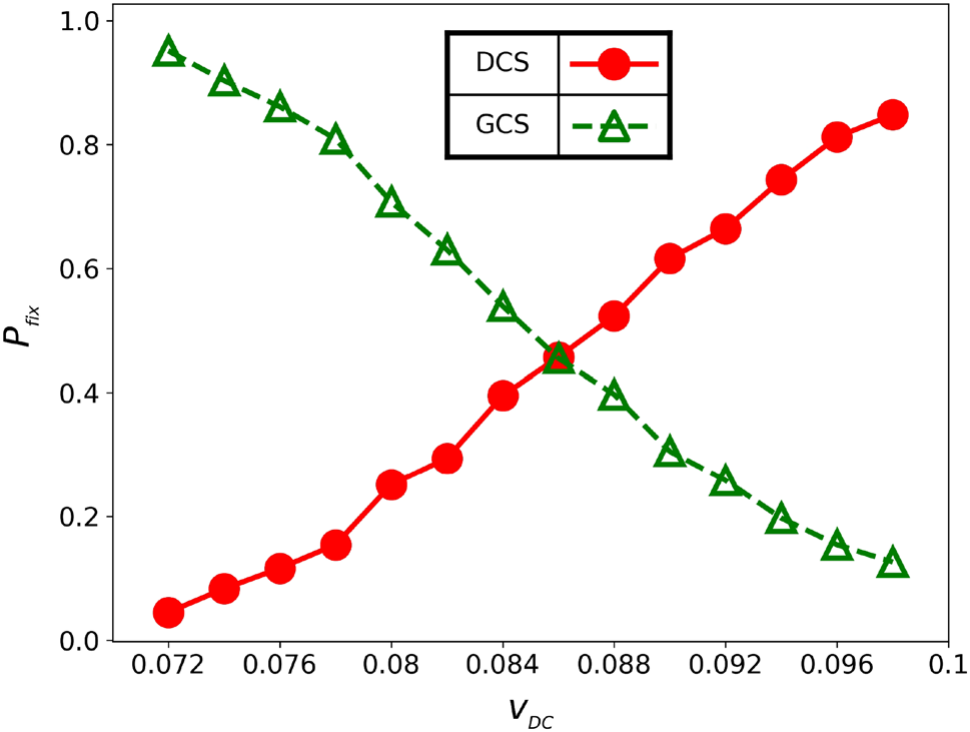
Fixation probability, $P_\text {fix}$, versus the speed of DCS chasers, $v_{_\text {D}}$. Green triangles represent fixation to GCS, while red circles represent fixation to DCS. The speeds of GCS chasers and targets are $v_{_\text {G}}= 0.07$ and $v_{_\text {T}}= 0.1$, respectively.

The GCS (DCS) fixation probability is defined as the number of the GCS (DCS) fixations divided by the total number of simulations. Figure 7 presents the comparison of fixation probabilities of the GCS (green triangles) and the DCS (red circles), at different values of the DCS chasers’ speed, $v_{_\text {D}}$. The results show that the GCS has a higher fixation probability compared to the DCS when $v_{_\text {D}}\le 0.084$. It may appear contradictory that our results for Model C indicate a higher probability of fixation for the GCS compared to the DCS, while in Model B, the DCS chasers have higher average payoffs than the GCS in heterogeneous sets when $v_{_\text {D}}\ge 0.08$, as shown in Fig. 5. Additionally, according to Fig. 6, more DCS chasers are expected to be present than GCS chasers in the “Nash Equilibrium” state, regardless of the number of chasers pursuing a target for $v_{_\text {D}}=0.082$.

## Summary and discussion

In summary, we have investigated the problem of group chase and escape by introducing two chasing strategies: One is to go directly to the nearest target, and the other is to surround a target together with other nearby chasers. We have discovered a social dilemma between these two strategies: That is, if we observe their performance separately, the latter clearly outperforms the former through cooperative hunting. However, if both types of chasers are running after a target, it is the direct chaser that usually benefits from the other chaser’s cooperation. The dilemma is overcome when cultural transmission is facilitated by learning: The proposed learning rule induces competition among strategies, whereas traditional evolutionary dynamics based on biological reproduction would naturally lead to individual selection that favors defection.

We could ask ourselves to which our mechanism belongs among the five representative ones for evolution of cooperation^43^. We have neither reciprocity or kin recognition in our dynamics, so the connection can be made to group selection. It is worth noting that the group structure in this study, dividing the whole population into sets, is not fixed but constantly changing, differently from the conventional setting for group selection^36^. Nevertheless, the inter-set interaction plays a crucial role. If a chaser were to learn the successful strategies only within its own set, the population would fix to DCS more often. However, our model allows chasers to learn from successful neighbors, even outside their set. We observe that allowing both intra-set and inter-set learning leads to a more prevalent fixation on GCS strategies. Notably, sets where GCS chasers are dominant tend to be more effective hunters compared to those dominated by DCS. Consequently, chasers learning from these neighboring sets often adopt the more successful GCS strategies, enhancing the wider adoption of GCS across sets. This process, effectively a form of group selection at the set level, tips the balance in favor of GCS strategies across the population. As a result, despite the tendency of DCS strategies to spread within their sets, the inter-set learning facilitates a broader propagation of GCS strategies. A critical aspect of cooperation evolution in our model is the parallel operation of group selection (between sets) and individual selection (within sets). Agents adopt strategies from nearby successful agents, ensuring rapid trait spreading both within and between sets and hence the “group selection” between sets works on a similar time-scale as the “individual” one within a set.

The social dilemma we examine is milder compared to the classic prisoner’s dilemma. Mapping GCS to Doves and DCS to Hawks, our model shares similarities with the Hawk-Dove or Chicken game. However, an essential difference exists: the conventional Chicken game revolves around a two-player conflict, whereas our model encompasses multiple players. This distinction becomes vital when we aim to elucidate the evolution of cooperation within a social dilemma framework. In our model, we have devised an extended version of the Chicken game. In this setting, the Nash equilibrium leads to a combination of DCS and GCS strategies, but not in an exact half-and-half proportion. The key finding of our work is that, through learning, the population can ultimately adopt GCS more than DCS, even though DCS appears more frequently than GCS in the Nash equilibrium.

Admittedly, the implicit assumption that the GCS and the DCS can be learned equally fast through observation could well be an oversimplification because the intellectual capacity required by the GCS in its strict sense would be enormous. It is also worth mentioning that cooperation and defection are not always simple choices but often coordinated activities that have to be learned over a long time span, even if it is shorter than a time scale of generations. Our viewpoint in this work is that the GCS is a mathematical substitute for more practical heuristics to surround a target without excessive cognitive loads, as has been proposed to explain wolf-pack hunting behavior^4^. In addition, the agent-based model utilized in this study may not entirely capture the complexities of actual predator-prey interactions, especially the dynamics of the evolution of chasing strategies in reality. Our model is parameter-heavy and includes a complicated step for assigning payoffs to different strategies.

Nevertheless, our model addresses the challenges for demonstrating cooperative evolution through group selection when the strategy update procedures and model parameters are not fine-tuned. By introducing learning from successful chasers in the surrounding environment, regardless of to which group the successful chasers belong, we demonstrate that cooperation can evolve without the need for explicit mechanisms such as direct or indirect reciprocity. This finding may cast new light on the ongoing efforts to construct a model incorporating group or multi-level selection.

## Data Availability

The simulation code can be accessed on GitHub at https://github.com/Li1221na/PredatorPreyModel. Additionally, a video of a single simulation for each model is available on YouTube at https://www.youtube.com/watch?v=lTGBAlLnQIY&ab_channel=PredatorPrey. Other datasets used and analyzed during this study are available from the corresponding author upon reasonable request.
